# Undergraduate ultrasound education at German-speaking medical faculties: a survey

**DOI:** 10.3205/zma001242

**Published:** 2019-08-15

**Authors:** Robert Wolf, Nicole Geuthel, Franziska Gnatzy, Daisy Rotzoll

**Affiliations:** 1 University of Leipzig, Faculty of Medicine, Skills and Simulation Centre LernKlinik Leipzig, Leipzig, Germany; 2 University Hospital Leipzig, Department of Paediatrics, Clinic for Paediatric Surgery, Leipzig, Germany; 3 St. Elisabeth Hospital Leipzig, Clinic for Internal Medicine II, Leipzig, Germany

**Keywords:** clinical skills, ultrasound education, medical students, curriculum development, peer-teaching

## Abstract

**Background:** The purpose of this study was twofold: to assess the status of undergraduate medical ultrasound (US) education in the German-speaking area and to suggest a possible framework for a longitudinal undergraduate medical US curriculum based on the study results and a literature review.

**Methods:** The survey included 44 medical faculties in the German-speaking area: 37 in Germany, four in Austria and three in German-speaking Switzerland. A standardized questionnaire focused on the following aspects of undergraduate medical US education: general information, organization, resources, assessment methods and evaluation.

**Results: **Data from 28 medical faculties were analysed. 26 out of 28 medical faculties offered US courses, 21 offered compulsory as well as elective courses, four offered compulsory and one elective courses only. 27 medical faculties supported US skills implementation. Abdominal US (n=25) was most common in teaching basic US skills. A learning objective catalogue was provided at 15 medical faculties. At 22 medical faculties, medical specialists were involved in undergraduate medical US education. 24 out of 26 medical faculties thought that peer-teaching is important to convey US skills. Medical faculties used the following methods to assess US skills: objective structured clinical examination (OSCE, n=7), non-standardized practical exams (n=4), non-standardized combined oral-practical exams (n=2) or direct observation of procedural skills (DOPS, n=1). 25 out of 26 medical faculties evaluated their US courses and 19 made suggestions for improvements in undergraduate medical US education.

**Conclusion: **Medical faculty members in the German-speaking area have recognized the relevance of undergraduate medical US education. So far, courses are offered heterogeneously with rather short hands-on scanning time and high student-instructor ratio. Based on the results of this study and a literature review we suggest a possible framework and milestones on the way to a longitudinal undergraduate medical US curriculum.

## Zusammenfassung

**Hintergrund:** Ziel dieser Studie war zum einen den derzeitigen Stand der Ultraschall-(US)-Ausbildung im Humanmedizinstudium im deutschsprachigen Raum zu überprüfen. Zum anderen sollte – basierend auf den Ergebnissen dieser Untersuchung und einer Literaturrecherche – eine mögliche Struktur eines longitudinalen US-Curriculums vorgeschlagen werden.

**Methoden:** Die Umfrage schloss 44 medizinische Fakultäten des deutschsprachigen Raumes ein: 37 in Deutschland, vier in Österreich und drei in der deutschsprachigen Schweiz. Ein standardisierter Fragebogen beinhaltete allgemeine Angaben, Organisation, Ressourcen, Überprüfung des Lernerfolgs und Evaluation der US-Ausbildung im Humanmedizinstudium.

**Ergebnisse: **Daten von 28 medizinischen Fakultäten wurden ausgewertet. 26 von 28 medizinischen Fakultäten boten US-Kurse an, davon 21 sowohl Pflicht- als auch Wahlkurse, vier ausschließlich Pflichtkurse und eine ausschließlich Wahlkurse. 27 medizinische Fakultäten befürworteten die Implementierung einer studentischen US-Ausbildung. Die Abdomensonographie galt bei 25 der befragten Fakultäten als beliebteste Methode, um grundlegende US-Fertigkeiten zu unterrichten. Ein Lernzielkatalog wurde an 15 medizinischen Fakultäten zur Verfügung gestellt. An 22 medizinischen Fakultäten waren FachärztInnen an der US-Ausbildung im Humanmedizinstudium beteiligt. 24 von 26 medizinischen Fakultäten fanden Peer-Teaching sinnvoll, um US-Fertigkeiten zu vermitteln. Als Prüfungsmodalitäten wurden „objective structured clinical examination“ (OSCE, n=7), nicht-standardisierte praktische Prüfungen (n=4), nicht-standardisierte kombiniert mündlich-praktische Prüfungen (n=2) oder „direct observation of procedural skills“ (DOPS, n=1) verwandt. 25 von 26 medizinischen Fakultäten gaben an, ihre US-Kurse zu evaluieren und 19 formulierten Verbesserungsvorschläge der US-Ausbildung im Humanmedizinstudium.

**Schlussfolgerung: **An medizinischen Fakultäten im deutschsprachigen Raum wird die Vermittlung von US-Fertigkeiten als relevant eingestuft. Bisher werden US-Kurse heterogen mit eher kurzen praktischen Übungszeiten und hohem Verhältnis von Studierenden zu Lehrenden angeboten. Basierend auf den Ergebnissen dieser Studie und einer Literaturrecherche schlagen wir sowohl eine mögliche Struktur eines longitudinalen Curriculums einer US-Ausbildung im Humanmedizinstudium als auch Meilensteine auf dem Weg dorthin vor.

## 1. Background

Ultrasound (US) as a diagnostic tool is used by almost every medical discipline today. Young residents are expected to have basic US skills and knowledge of the method. However, residents report they have been taught in US only to a limited degree [1]. The national competency-based learning objective catalogues of Austria [2], Germany [http://www.nklm.de] and Switzerland [3] recommend only theoretical knowledge and a “knows how” competence level [4] of US skills. Nevertheless, medical students acquire basic US skills and reach a competence level of “shows how” when instructed by a medical specialist or trained peer-student-tutor offering appropriate hands-on scanning time and small-group training [5], [6], [7].

Several international medical faculties have implemented US course components into their core curriculum [8], [9], [10], [11], [12], [13], [14], [15], [16]. In 2011, Hoppmann et al. integrated a vertical US curriculum at the University of South Carolina, School of Medicine for all medical students across four years of medical school [8].

In the German-speaking area the medical faculties of Duesseldorf and Muenster offer a basic US curriculum for all medical students (of one study year) with hands-on training sessions over 10 weeks with 1.5 to 2 hours per session and small group sizes with three to five medical students [17], [18]. Baltarowich et al. developed a two-folded national US curriculum for medical students in the United States. They promoted the utilization of US as a teaching tool during preclinical and as a basic examination skill during clinical education [19]. In 1996, at the Medical Faculty of Hannover, Teichgräber et al. first described the educational advantages of US within the preclinical curriculum to teach gross anatomy [16]. Meanwhile, similar programmes have been implemented by numerous educators [20], [21], [22], [23], [24], [25]. However, in a previous study by Lohmann et al., surveying the postgraduate US medical education at German university hospitals, only 50% of medical US specialists thought that the integration of US into the gross anatomy curriculum would be useful [26]. 

Nevertheless, medical students have recognized that “It is time for the sonoscope” [27] and founded student initiatives such as “Sono4You”. “Sono4You” was founded in Vienna in 2007 and is now present in different European cities with an expanding amount of course offers [http://www.sono4you.org/ accessed 07 Jun 2019]. There are partnerships to other student initiatives (e.g. “Sono4Students” Bonn [https://sono4students.uni-bonn.de/ accessed 07 Jun 2019], “AG Sonografie” Berlin [https://lernzentrum.charite.de/leistungen/arbeitsgruppen/ag_sonografie/ accessed 07 Jun 2019]), as well as to several skillslabs in the German-speaking area and to the national US societies of Germany, Switzerland and Austria (DEGUM [https://www.degum.de/index.html accessed 07 Jun 2019]; SGUM [https://sgum-ssum.ch/ accessed 07 Jun 2019]; OEGUM [http://www.oegum.at/ accessed 07 Jun 2019]).

The European Federation of Societies for Ultrasound in Medicine and Biology (EFSUMB) recently promoted undergraduate medical US education within European medical faculties and formulated steps to achieve this goal [28].

Following this rapid development and the controversial views on the subject, it is necessary to understand how and to which extent US is taught at German-speaking medical faculties. The purpose of this study was therefore twofold: to assess the status of undergraduate medical US education in the German-speaking area and to suggest a possible framework for a longitudinal undergraduate medical US curriculum based on the study results and a literature review.

## 2. Methods

### 2.1. Questionnaire

To evaluate the status quo of undergraduate medical US education in the German-speaking area the questionnaire by Beckers et al. was adapted [29]. This questionnaire surveyed the status of German undergraduate medical education in emergency care medicine in 2009 with its relevant components. This survey was developed by the “Committee for Emergency Medical Care and Simulation” within the “German Association for Medical Education” (GMA) [https://gesellschaft-medizinische-ausbildung.org/ausschuesse/notfallmedizin-und-simulation.html accessed 07 Jun 2019]. 

The questionnaire (see attachment 1 ) focused on the following aspects of undergraduate medical US education:

1. General information
2. Organization
3. Resources
4. Assessment methods
5. Evaluation

In total, 32 questions were posed in closed-ended (n=16), open-ended (n=3) and multiple-response format (n=13).

#### 2.2. Participating medical faculties

In December 2015, 44 medical faculties were listed for the German-speaking area on the websites of “Medizinischer Fakultätentag” [https://medizinische-fakultaeten.de/ accessed 07 Jun 2019] and “Thieme via medici informieren” [https://www.thieme.de/viamedici/vor-dem-studium-infos-zum-medizinstudium-1493/a/medizinische-fakultaeten-22820.htm accessed 07 Jun 2019]: 37 in Germany, four in Austria and three in German-speaking Switzerland.

#### 2.3. Time frame

All skillslabs in Germany, Switzerland and Austria were contacted via the “Skillslab-Forum” in December 2015 – a communication platform for all skillslabs in the German-speaking area, supervised by the University of Cologne, Faculty of Medicine [https://lists.uni-koeln.de/mailman/listinfo/skillslab-forum/ 07 Jun 2019]. An information leaflet (see attachment 2 ) gave an overview of the survey. The questionnaire could either be filled out via a protected word- or pdf-document. Due to the initial low response rate, representatives of the undergraduate medical US education were contacted via the skillslab director, dean’s office or internet search of the medical faculty’s website. All participants were reminded every two weeks via e-mail or telephone call. A final reminder was sent by postal mail with an enclosed, self-addressed and stamped envelope. The final response rate in May 2016 was 64% (28/44). 

First results were presented at the “XI. International Skillslab Symposium in Essen” [30] and the “40. Dreiländerteffen of DEGUM, SGUM and OEGUM” [31]. 

#### 2.4. Data analysis

The answers of the closed-ended and multiple-response questions were numbered, pre-coded and typed into a SPSS data sheet (IBM Statistics SPSS20®, IBM Chicago). Answers of the three open-ended questions were assigned to keywords and then pre-coded equivalent to the answers of the multiple-response and closed-ended questions. Contact data of participants and US representatives were listed in a separate data sheet to keep the analysis anonymous. Data on course duration were fragmentary and varied from location to location. If time specifications could not be confirmed by internet search, they were excluded from data analysis.

## 3. Results

### 3.1. General information 

26 out of 28 participating medical faculties offered US courses, 21 offered compulsory as well as elective courses, four offered compulsory and one elective courses only.

27 medical faculties supported US skills implementation into undergraduate medical curricula. Supportive opinions are shown in table 1 (Tab. 1). Arguments against undergraduate medical US education were that performing US is a highly specialized clinical skill and that US skill acquisition is not part of the German, Swiss or Austrian competency-based learning objective catalogue. Additionally, the input to teach US would outweigh the output because it should be practiced daily.

The person in charge of the undergraduate medical US education differed extremely between the medical faculties. Clinical (n=8) as well as skillslab staff (n=6), peer-student-tutors (n=4), the director of the US unit (n=3) and deans (n=2) were responsible. Three medical faculties could not specify or had no personnel for teaching US. 

#### 3.2. Organization 

Budget for the undergraduate medical US education was either forwarded by the medical faculty (n=14), a department of the university hospital (n=13) or the dean’s office (n=12). Third-party funds (n=5), government grants (n=5) or student representative funds (n=1) were also included. 17 medical faculties reported at least two or more financial aids.

US courses were offered as single courses at 11 medical faculties whereas ten medical faculties used a longitudinal curriculum approach. 16 medical faculties reported elective US courses either instead or as an add on to compulsory US courses.

Especially internal medicine (n=20), radiology (n=17) or both were involved in undergraduate medical US education. Family medicine was involved at seven, surgery at six, gynaecology at four, anaesthesiology, paediatrics, urology and anatomy at two and otorhinolaryngology, neurology and dermatology at one medical faculty, respectively. 

11 medical faculties followed standards of the EFSUMB or the national US societies of Germany, Austria or Switzerland. A web presence of the undergraduate medical US education existed at 11 medical faculties. Medical students could prepare for courses with a course book/ script (n=15), e-learning modules (n=10), lectures (n=8) or independent US skills training facilities (n=2).

**Figure 1:**
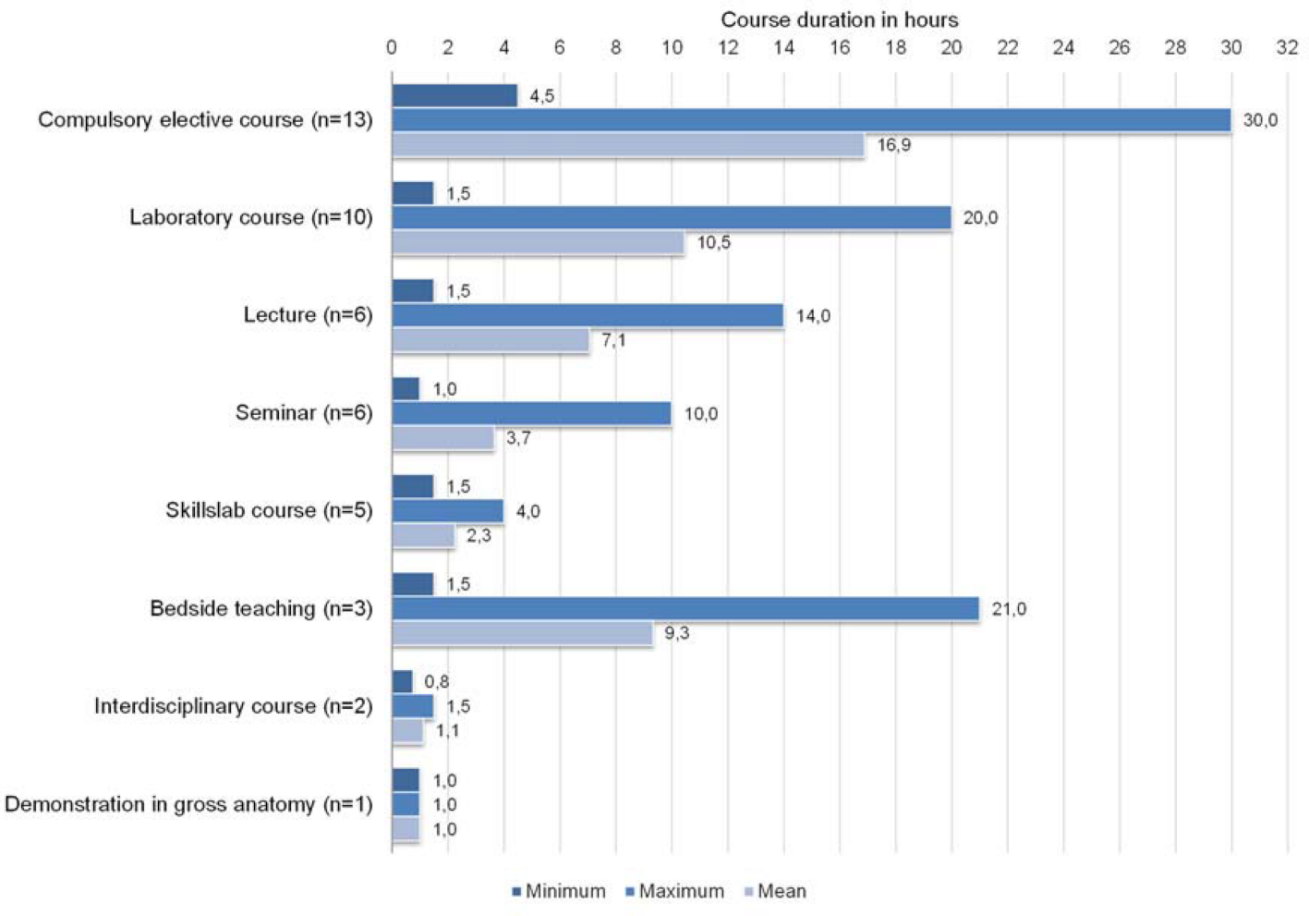
Course duration of compulsory US courses.

Compulsory US courses were offered by 25 medical faculties mainly as compulsory elective courses and laboratory courses during clinical training (3rd to 5th year of medical study, see table 2 (Tab. 2)). Every medical faculty in the German-speaking area offered a number of compulsory elective courses and medical students were required to select one during preclinical and one during clinical training [32]. Course durations of compulsory US courses are displayed in figure 1 (Fig. 1). Group sizes ranged from three to 60 medical students (ten to 240 within lectures) with one or two instructors. 

Elective US courses were offered by 22 medical faculties mainly as skillslab courses during clinical training (3rd to 5th year of medical study, see table 2 (Tab. 2)). Course duration of elective US courses ranged from a single 90-minute-course to a 42-hours-course-programme. Group sizes ranged from three to 15 medical students with one to three instructors. 

Medical students examined each other (n=25), but also simulators (n=10), patients (n=7) or instructors and peer-student-tutors (n=3) were stated as objects of US examinations. Abdominal US skills training was most frequently implemented. Table 3 (Tab. 3) gives an overview of examined organs and organ systems in US skills training. 

A learning objective catalogue for undergraduate medical US education was provided at 15 faculties. Table 4 (Tab. 4) shows learning objectives of undergraduate medical US education and “knobology” (instruction of control panels of US machines, see also Hofer’s book “Sono Grundkurs” [33]).

#### 3.3. Resources

Especially medical specialists (n=22) or residents (n=12) were involved in undergraduate medical US education. At 12 medical faculties, instructors were certified by the national US societies. 18 medical faculties used their own didactic training and seven medical faculties had instructors with a Master of Medical Education degree. 

Peer-student-tutors played an increasing role not only in elective, but also in compulsory US courses. Six medical faculties offered special training for peer-student-tutors with a 4-week elective in the US unit or instruction sessions by medical specialists. Peer-teaching was thought to be important for undergraduate medical US education (n=24). Supportive opinions for peer-teaching are shown in table 5 (Tab. 5). 

A minimum of one and a maximum of 12 US machines (mean: 5) were available for undergraduate medical US education. 

#### 3.4. Assessment methods

Medical faculties used the following methods to assess US skills (see table 6 (Tab. 6)): written exams (n=9), objective structured clinical examination (OSCE, n=7), non-standardized practical exams (n=4), non-standardized combined oral-practical exams (n=2), oral exams (n=1), essays (n=1) or direct observation of procedural skills (DOPS, n=1). Eight medical faculties had no assessment implemented.

#### 3.5. Evaluation

US courses were evaluated by 25 medical faculties either online, paper-based or orally. Evaluations were published by 15 medical faculties. 19 medical faculties made suggestions for possible curriculum improvements (see table 7 (Tab. 7)).

## 4. Discussion

### 4.1. Have German faculty members recognized the relevance of undergraduate medical US education?

Medical students are able to acquire basic US skills and a “shows how” competence level of US skills which they can build on during residency [4], [5], [34]. Syperda et al. suggested approximately ten hours of theoretical preparation and 40 hours of practical US courses to train second-year medical students in basic US skills, when measuring their post-training results [5]. In the German-speaking area only few medical faculties (e.g. Duesseldorf, Muenster and Giessen) offered compulsory US courses with a comparable amount of hands-on scanning time (up to 20 hours) for all medical students of one study year [17], [18]. The overall duration of compulsory US courses differed considerably from location to location (total: one to 30 hours, mean: one to 17 hours). Instead, a variety of elective skillslab courses and compulsory elective courses were offered during clinical training to supply hands-on training time for only a limited number of medical students. 

Student-instructor ratio in compulsory US courses ranged from 3:1 to 60:1 (excluding lectures). Heinzow et al. introduced group sizes of three medical students in hands-on training sessions due to medical students’ evaluations [18]. Hofer et al. implemented courses where five medical students were instructed by one peer-student-tutor together with one medical specialist rotating between four different groups twice a day. Consequently, only four medical specialists per week were needed to provide courses for 160 medical students per semester and 320 per study year [17]. The ideal student-instructor ratio should not be higher than 5:1 [17], [18], [19], but Tolsgaard et al. recently found that instruction of student pairs (“dyad practice”) actually improved the efficiency of training and was not inferior to individual instruction [6]. Therefore, group sizes of six medical students seem suitable as well.

The importance of peer-student-tutors and their benefits for small-group learning sessions was widely recognized. Peer-student-tutors are not inferior to medical faculty members in teaching medical students basic US skills [35]. Nevertheless, when it comes to interpreting physiological and pathological findings peer-student-tutors may be inferior in teaching due to lack of experience [36]. Consequently, peer-student-tutors need to be trained intensely. The curriculum for peer-student-tutors published by Hofer et al. consisted of four steps: working as dissecting-class tutor in gross anatomy, a 4-week elective in the US unit, role-play courses with experienced US peer-student-tutors and didactic seminars [17]. A similar curriculum by Celebi et al. involved a 2-hour seminar, a 3-week full-time elective in an US unit, a 2-hour supervised session with simulation and a 12-hour standardized peer-student-tutor didactic seminar [35].

Internal medicine and radiology were most frequently involved in undergraduate medical US education. Abdominal US is commonly performed by internists and radiologists in clinical routine and at the same time popular in teaching medical students basic US skills [26]. Adding other medical disciplines leads to a variety of potential US-based courses that could be integrated into bedside teaching during clinical years of medical education [19]. Medical faculties reported to offer such practical US courses during bedside teaching, but number and content of these courses were hardly measurable. Hoppmann et al. established a vertical curriculum that provides US experience over four years of medical school. During rotations in different clinical subjects, medical students had the chance to experience US through discipline-related hands-on training sessions [8], [37].

Medical faculties in the German-speaking area (e.g. Bochum, Essen, Hannover and Leipzig) used the educational advantages of US within the preclinical curriculum to teach gross anatomy, which had first been described by Teichgräber et al. in 1996.

Only a third of participating medical faculties introduced standardized practical skills exams, OSCE or DOPS, to assess US skills. Hofer et al. introduced multiple organ-specific OSCE exam stations [38]. DOPS introduced by Heinzow et al. grades the examiner’s abilities using seven different items [18]. Both can be considered as suitable for assessing US skills acquisition in a standardized, reproducible and objective format and should be therefore favoured over other, non-standardized assessment methods. 

Medical faculty members in the German-speaking area have recognized the relevance for undergraduate medical US education, but courses are offered heterogeneously with rather short hands-on scanning time and high student-instructor ratio. Similar findings were reported by Bahner et al. in the United States [39] and Steinmetz et al. in Canada [40]. To state it with the words of Badea et al.: “There is an obvious lack of vision on this aspect even though there is a consensus regarding the value of ultrasonography in the teaching of various disciplines (anatomy, physiology, physiopathology and semiology) as well as in completing the diagnosis algorithm (clinical applications in different specialties and medical emergencies)” [11]. 

#### 4.2. What steps must be taken on the way to a longitudinal undergraduate medical US curriculum?

**Figure 2:**
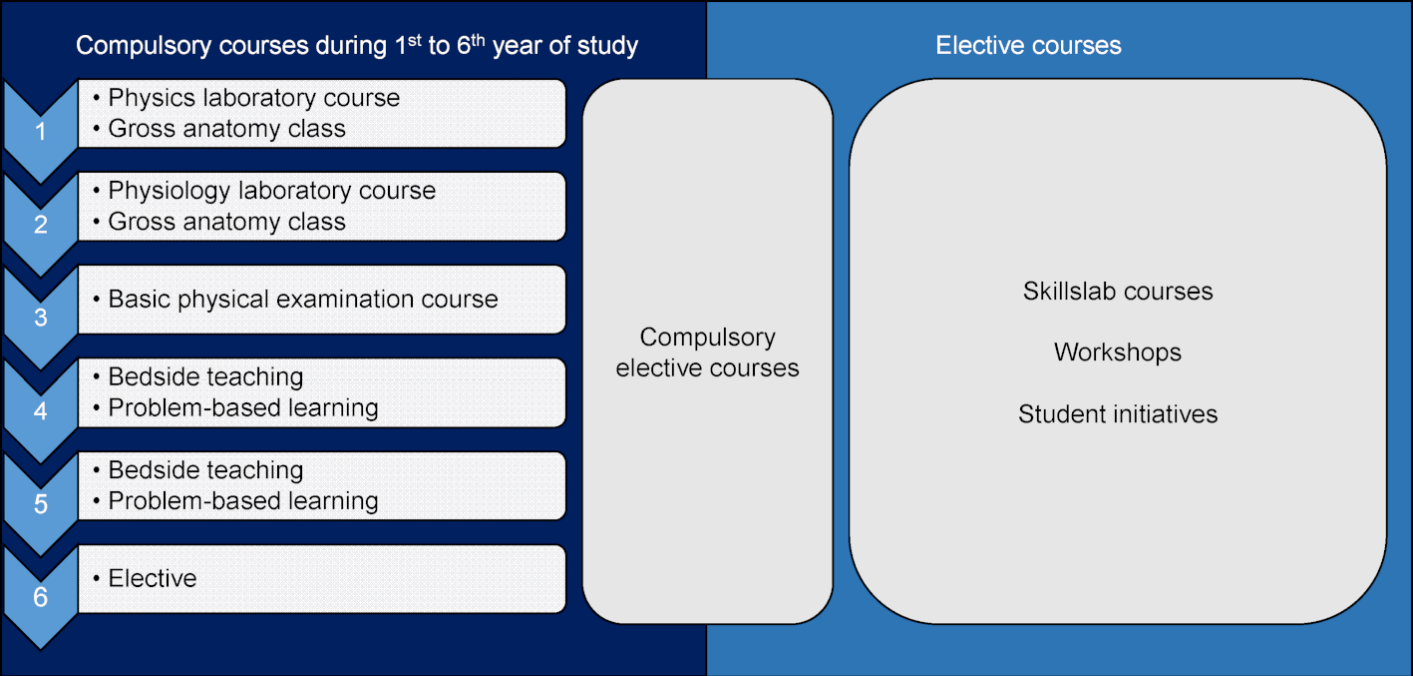
Possible integration points of undergraduate medical US education in a traditional German study program.

Obviously, there cannot be one solution for an undergraduate medical US curriculum for every medical faculty in the German-speaking area. Each medical faculty has its own resources and starts from different levels in developing its own undergraduate medical US curriculum. Nevertheless, minimum standards for the integration of undergraduate medical US education are needed to overcome competing interests and to encourage the dialogue between medical specialists, medical faculty members, deans and medical students. Based on the results of this study and a literature review [17], [18], [19], [28], [36], [41] we suggest a possible framework and the following milestones on the way to a longitudinal undergraduate medical US curriculum:

1. Implement US at different stages throughout the curriculum. Possible integration points of US in a traditional study programme are:

- e.g. gross anatomy course, physiology or physics laboratory course during preclinical years of study
- e.g. basic physical examination course, problem-based learning, bedside teaching or elective during clinical years of study
- e.g. compulsory elective courses, skillslab courses, workshops or courses provided by student initiatives during all years of study (see also figure 2 (Fig. 2))

1. Understand US as part of almost every medical discipline to teach its many possible applications.
2. Implement compulsory courses to supply every medical student with basic theoretical and practical US skills, rather than adding elective courses for only a limited number of medical students.
3. Add elective courses as extra US practice.
4. Gain for longitudinal US course implementation to increase total hands-on scanning time (approximately 20-40 hours in total).
5. Give a short theoretical introduction to each practical US session and/or link it to a lecture.
6. Network with your adjacent university hospital, medical faculty, dean’s office and skillslab as well as non-local medical faculties, skillslabs, student initiatives and national US societies.
7. Train peer-student-tutors or medical faculty members to supply hands-on scanning time for small groups (maximum of six medical students per group).
8. Ensure supervision by a medical specialist when examining human models to differentiate between physiological and pathological findings.
9. Let medical specialists rotate between study groups to decrease their workload.
10. Supply a learning objective catalogue for medical students.
11. Evaluate your US courses to improve local undergraduate medical US education.
12. Apply for additional financial support (e.g. support from dean’s office and medical faculty, third-party funds) and increase your chances by adding evaluation results.
13. Introduce suitable, standardized assessment methods (e.g. OSCE, DOPS).
14. Adjust the US skills competency level from “knows how” to “shows how” within the national competency-based learning objective catalogues.

#### 4.3. Limitations of this study

The responsibility for the undergraduate medical US education differed tremendously between faculties or could not be specified at all. The enclosure of three open-ended questions provided a wide range of personal opinions, but the need for categorizing answers for data analysis may have led to information loss. Time range and group sizes of compulsory and elective course components were answered very heterogeneously by medical faculties. 

Three years have passed since the survey has been carried out and the results have been published.

Therefore, another study is needed to describe the newest developments.

## 5. Conclusion

The next step is to integrate existing elective course offers into the curriculum to ensure practical US skills experience for all medical students. Networking with the own university hospital, medical faculty, dean’s office and skillslab as well as non-local medical faculties, skillslabs, student initiatives and national US societies can help in achieving this goal. Ultimately, a longitudinal undergraduate medical US curriculum during all years of study and the adjustment of the national competency-based learning objective catalogues are needed to establish US as a crucial examination skill for every future doctor.

## Abbreviations

- AG: “Arbeitsgemeinschaft”, Working group;
- DEGUM: German Society for Ultrasound in Medicine;
- DOPS: Direct observation of procedural skills;
- EFSUMB: European Federation of Societies for Ultrasound in Medicine and Biology;
- GMA: German Association for Medical Education;
- OEGUM: Austrian Society for Ultrasound in Medicine;
- OSCE: Objective structured clinical examination;
- SGUM: Swiss Society for Ultrasound in Medicine;
- US: Ultrasound.

## Data

Data for this article are available from the Dryad Digital Repository: http://dx.doi.org/10.5061/dryad.5rk56gg [42]

## Notes

### Ethics approval and consent to participate 

The goal of this study was obtaining a detailed description of the undergraduate US education of the German-speaking medical faculties. Consequently, the ethics committee of the Medical Faculty of Leipzig waived the requirement for an ethical approval procedure. The study was conducted in accordance to the declaration of Helsinki, revised form, Seoul 2008. All institutions gave consent to publish questionnaire results. The study participation was voluntary.

#### Authors’ contribution

RW adapted the questionnaire, performed data collection and analysis as well as drafted the manuscript. DR was involved in the design of the study and revised the manuscript. NG and FL implemented compulsory US skills courses and revised the manuscript. All authors read and approved the final manuscript. 

## Acknowledgements

The authors would like to thank all involved physicians, medical faculty members and medical students who volunteered to complete the questionnaire accurately. 

## Competing interests

The authors declare that they have no competing interests. 

## Supplementary Material

Quantitative and qualitative situation analysis of the undergraduate ultrasound education at medical faculties in the German-speaking area

Information leaflet

## Figures and Tables

**Table 1:**
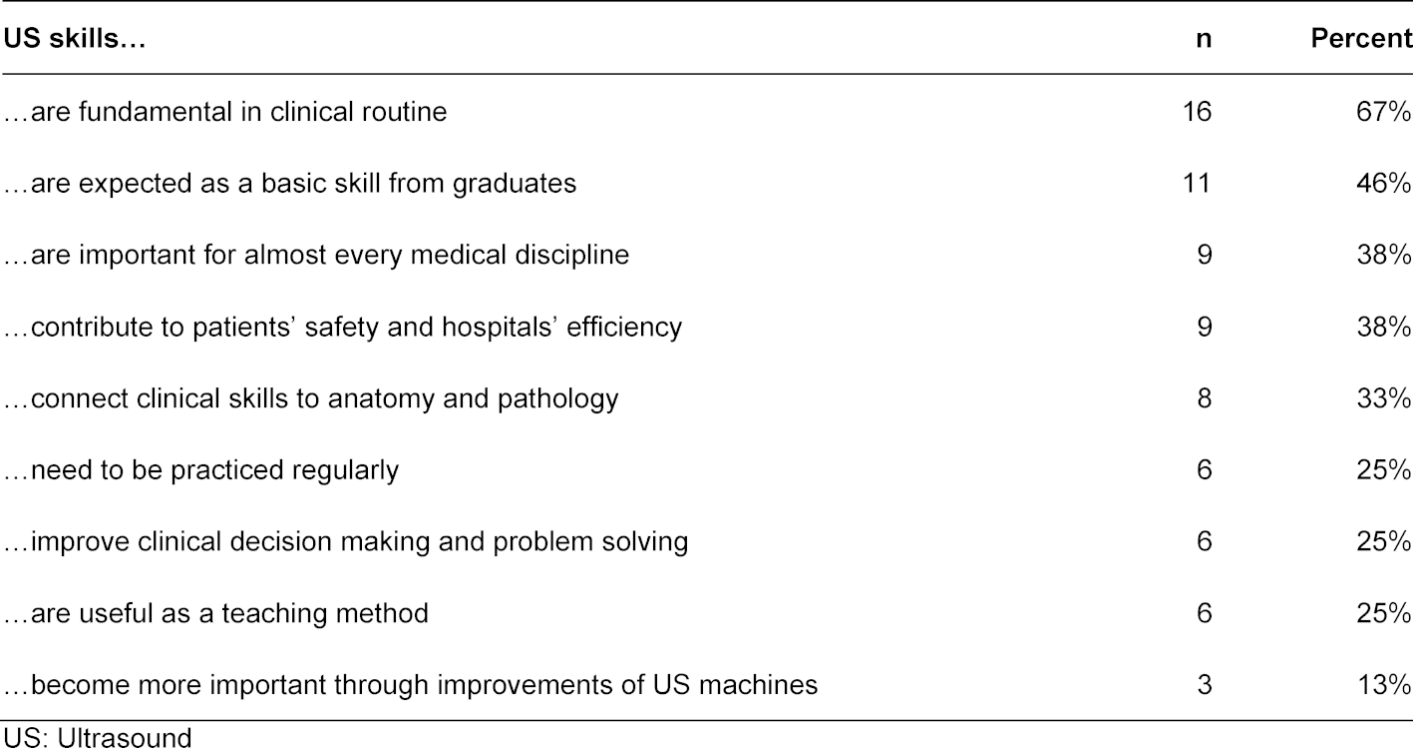
Support for US skills implementation in undergraduate medical curricula, n=24 (see question 5 of the questionnaire, open-ended)

**Table 2:**
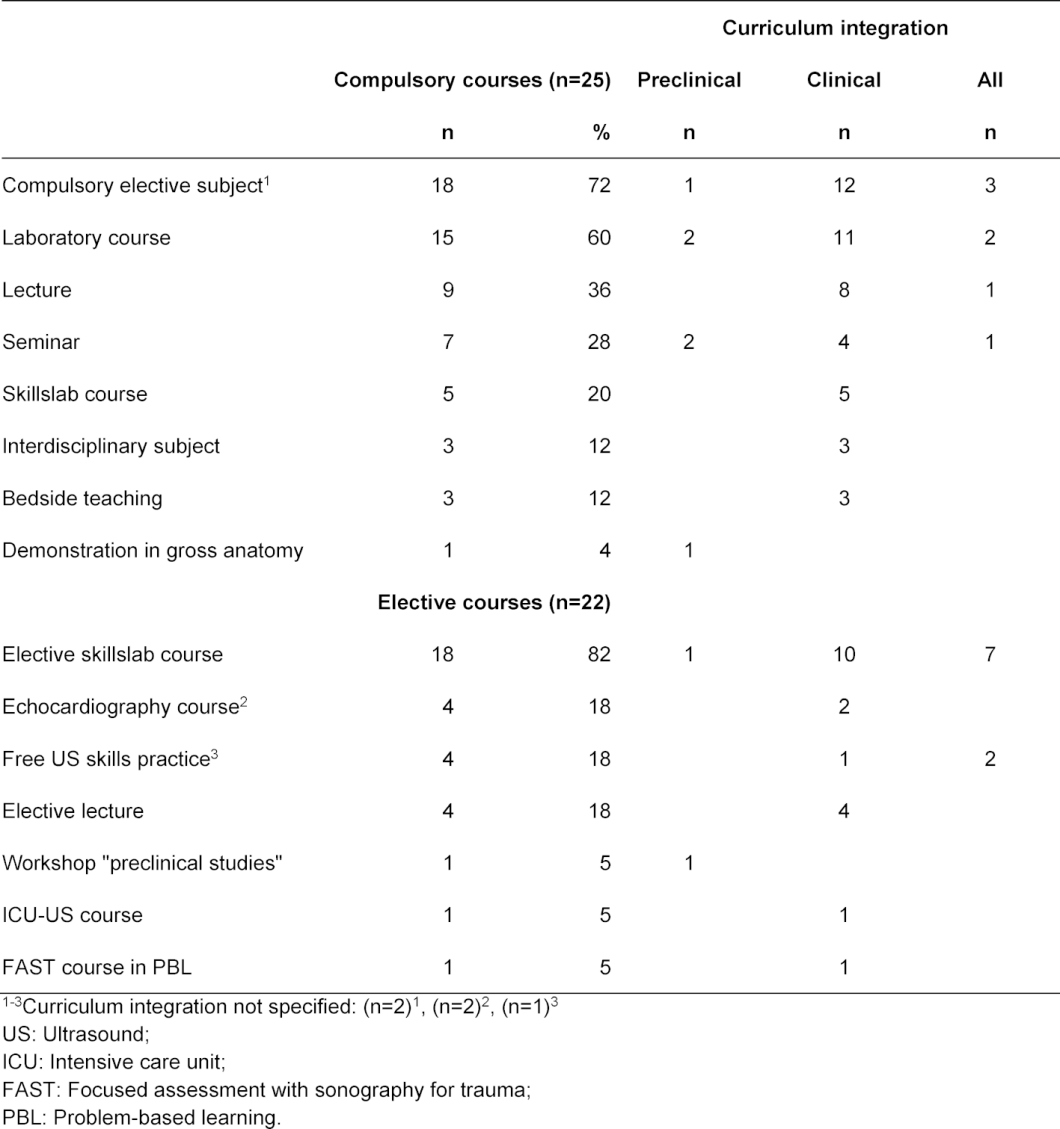
Integration of US skills courses into the curriculum (see question 15 and 16 of the questionnaire, multiple-response)

**Table 3:**
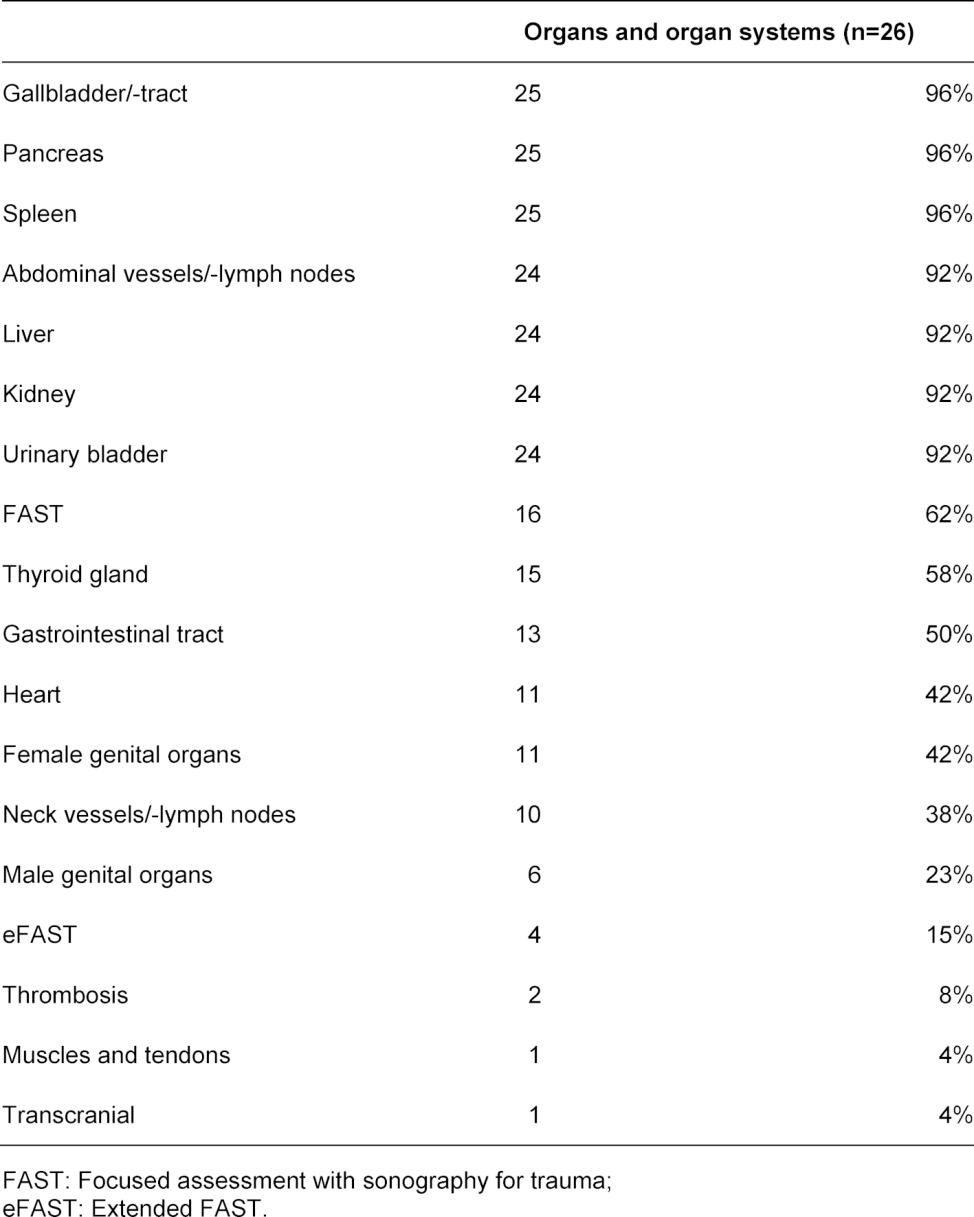
Examined organs and organ systems (see question 18 of the questionnaire, multiple-response)

**Table 4:**
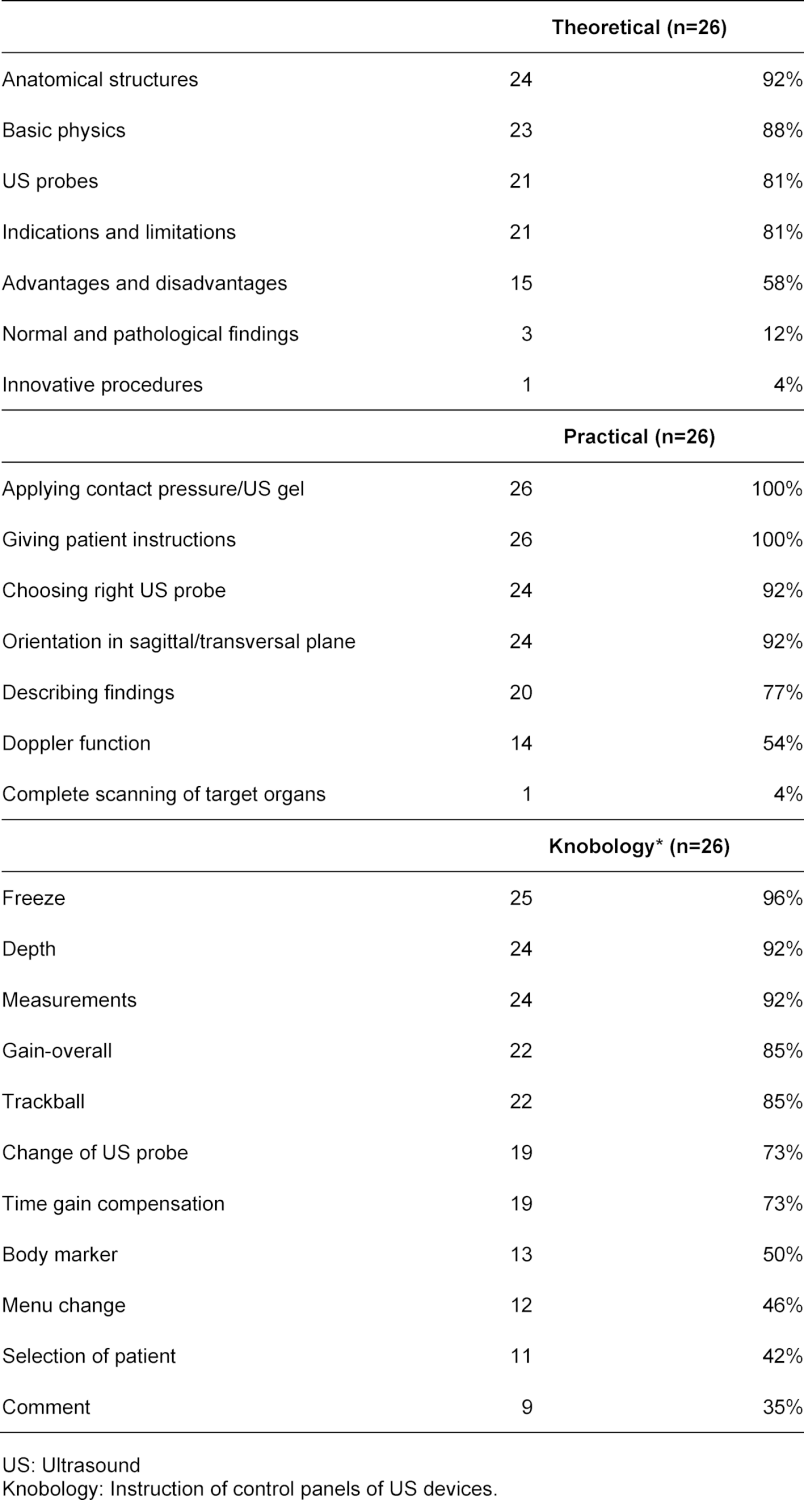
Learning objectives of undergraduate medical US education and US knobology (see questions 19 and 20 of the questionnaire, multiple-response)

**Table 5:**
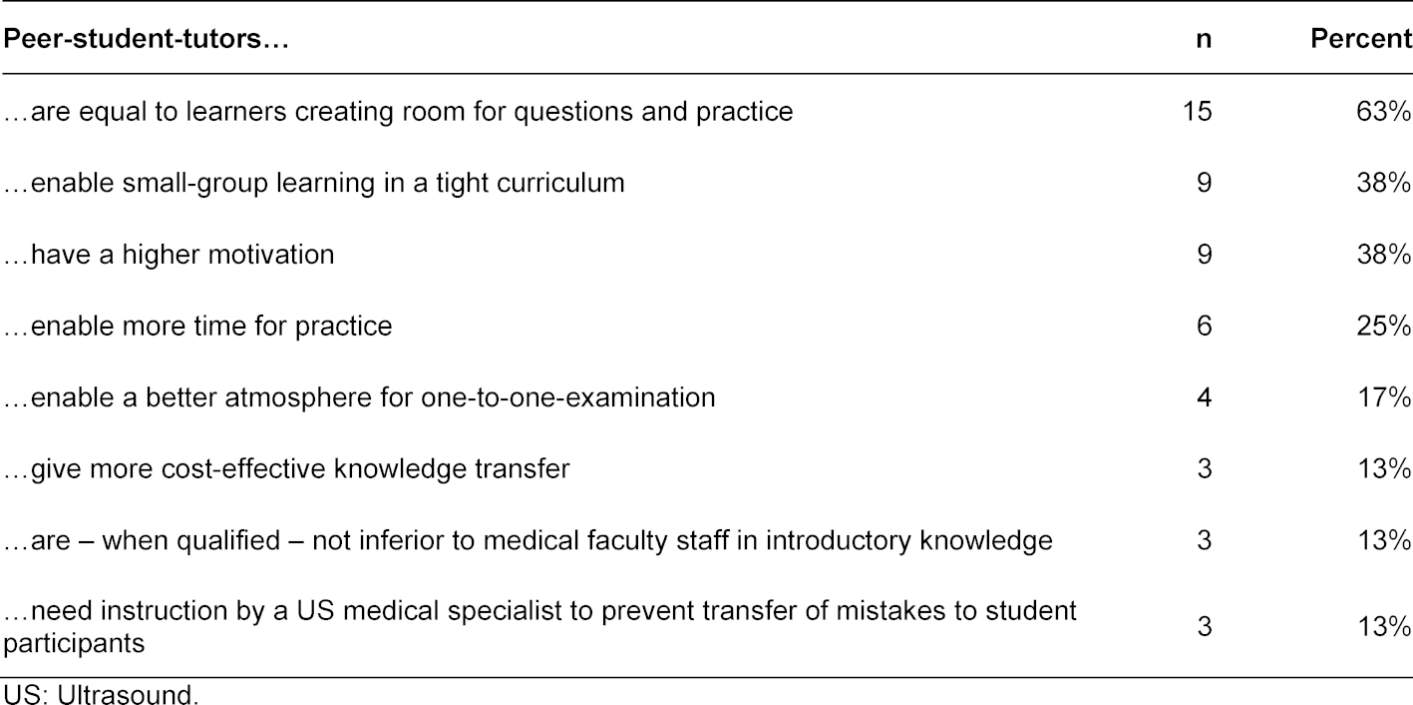
Support for peer-teaching in undergraduate medical US education, n=24 (see question 26 of the questionnaire, open-ended)

**Table 6:**
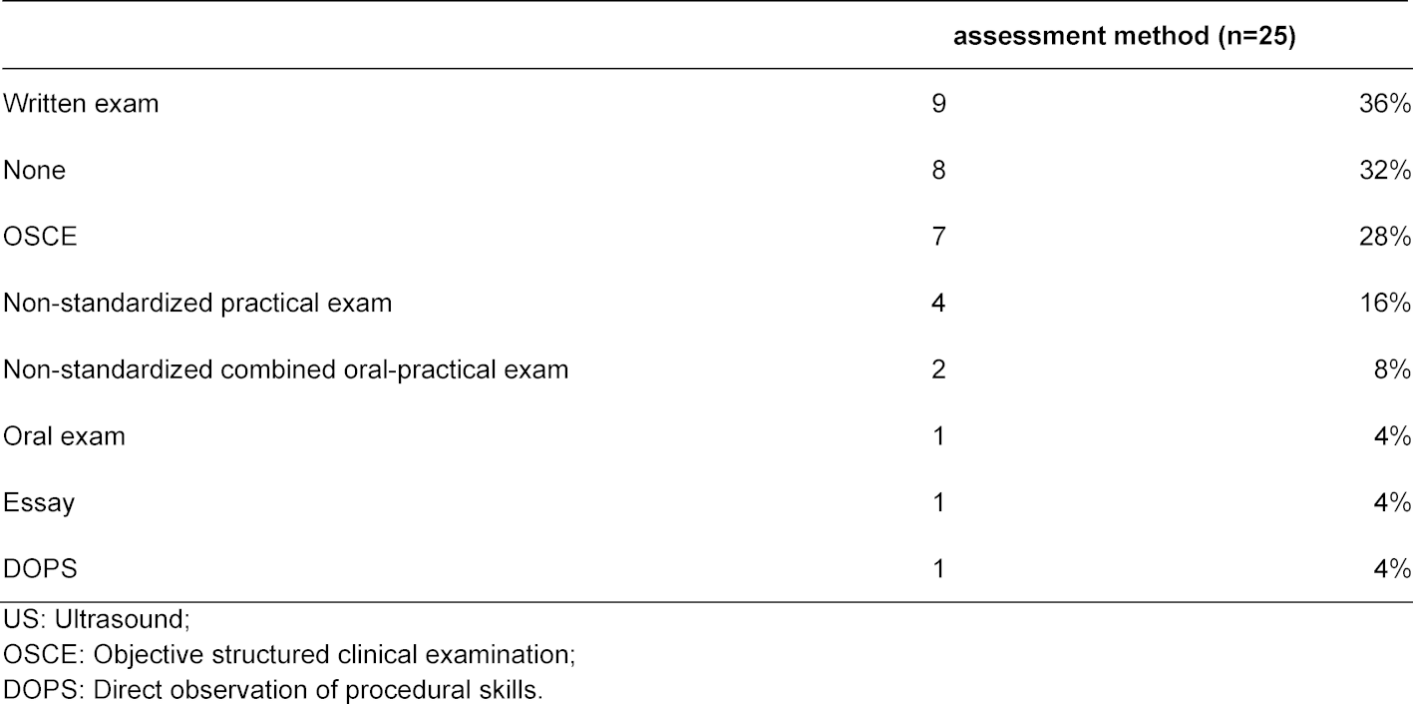
Assessment methods of the undergraduate medical US education (see question 27 of the questionnaire, multiple-response)

**Table 7:**
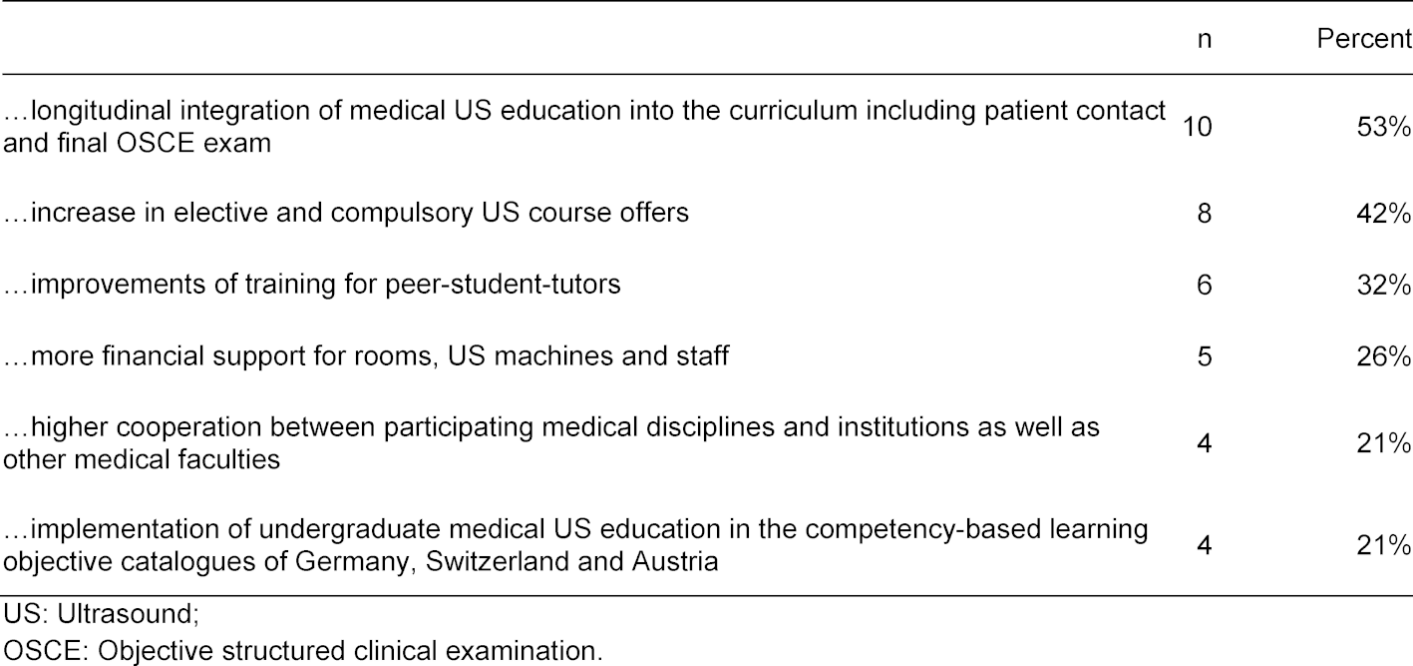
Suggestions for possible curriculum improvements of undergraduate medical US education, n=19 (see question 32 of the questionnaire, open-ended)
