# Supplementary material for: Undergraduate ultrasound education at German-speaking medical faculties: a survey
Source: GMS J Med Educ. 2019 Aug 15;36(4):Doc34. doi: 10.3205/zma001242 (PMC6737263; doi:10.3205/zma001242)
Supplement: Quantitative and qualitative situation analysis of the undergraduate ultrasound education at medical faculties in the German-speaking area [file JME-36-4-34-s-001.pdf]

Quantitative and qualitative situation analysis of the undergraduate ultrasound education at medical faculties in the German-speaking area

General information

Skillslab/ Medical Faculty:
Person responsible for ultrasound skills education:
E-mail:
Phone:

1. How is the study program organized?

☐ Traditional German medical curriculum ☐ Reformed study program

☐ Other (Please name!) ________________________________

2. How is the study programme structured?

☐ Semester ☐ Trimester ☐ Study year

3. Are ultrasound courses offered at your medical faculty /your skillslab?

☐ Yes ☐ No ☐ Not applicable

4. Do you think that ultrasound skills education for medical students is useful?

☐ Yes ☐ No ☐ Not applicable

5. Why do you think that ultrasound skills education for medical students is useful/not useful?

6. Who manages/organizes the ultrasound skills education?

Person responsible for ultrasound skills education:
E-mail:
Phone:

7. How is the ultrasound skills education financed? *(Multiple responses possible!)*

☐ Department of the university hospital ☐ Medical Faculty ☐ Dean's office

☐ Third-party funds ☐ Other (Please name!) ______________________________

Organization

8. How is the ultrasound skills education integrated into the curriculum?

☐ Single unit ☐ Longitudinal

☐ Other (Please name!) ______________________________

9. Which medical disciplines[1] are involved in the ultrasound skills education?
(Multiple responses possible!)

☐ Family medicine ☐ Anaesthesiology ☐ Surgery

☐ Gynaecology ☐ Ear, Nose and Throat (ENT) ☐ Internal medicine

☐ Neurosurgery ☐ Neurology ☐ Ophthalmology

☐ Paediatrics ☐ Radiology ☐ Urology

☐ Not applicable ☐ Other (Please name!) ______________________________

10. Does a learning objective catalogue for the ultrasound skills education exist?

☐ Yes ☐ No ☐ Not applicable

11. If yes, is it handed out to the students?

☐ Yes ☐ No ☐ Not applicable

12. Which standards regarding ultrasound are represented?

☐ German Society for Ultrasound in Medicine (DEGUM) ☐ European Federation of Societies for Ultrasound in Medicine and Biology (EFUSMB) ☐ No standard

☐ Other (Please name!) ____

[1] Adapted from sections of the DEGUM (www.degum.de).

13. How can students prepare for the ultrasound courses? *(Multiple responses possible!)*

☐ Course book ☐ E-Learning ☐ Lecture

☐ Not applicable ☐ Other (Please name!) ________________________________

14. Is there a website for the ultrasound skills education?

☐ Yes ☐ No ☐ Not applicable

If yes, please name! ____

15. How often and to what extent are <u>compulsory ultrasound</u> courses offered?
(Multiple responses possible!)

Description of the course *(If not listed, please use empty cells below!)*	Is offered during semester(s):	Number of credit hours[2]	Number of students per group	Number of instructors per group
☐ Lecture				
☐ Seminar				
☐ Interdisciplinary course				
☐ Laboratory course				
☐ Bedside teaching				
☐ Skillslab course				
☐ Compulsory elective course				
☐				
☐				

[2] Alternatively, you can display course duration in periods of 45 min, please state!

16. How often and to what extent are elective ultrasound courses offered?
(Multiple responses possible!)

Description of the course *(If not listed, please use empty cells below!)*	Is offered during semester(s):	Number of credit hours[3]	Number of students per group	Number of instructors per group
☐ Lecture				
☐ Seminar				
☐ Skillslab course				
☐				
☐				
☐				

17. What serves as object of ultrasound examination? *(Multiple responses possible!)*

☐ Patient ☐ Simulated patient ☐ Participant/student

☐ Simulator ☐ Instructor

☐ Other (Please name!) ____

18. Which organs-/ organ systems[4] are examined? *(Multiple responses possible!)*

☐ Thyroid gland ☐ Neck vessels/-lymph nodes ☐ Heart

☐ Abdominal vessels/-lymph nodes ☐ Liver ☐ Gall bladder/-tract

☐ Pancreas ☐ Spleen ☐ Gastrointestinal tract

☐ Kidney ☐ Urinary bladder ☐ Female genital organs

☐ Male genital organs ☐ Focused Assessment with Sonography for Trauma (FAST) ☐ Muscles and tendons

☐ Other (Please name!) ____

[3] Alternatively, you can display course duration in periods of 45 min, please state!

[4] Hofer, Matthias: Sono Grundkurs, pg. 4-5.

19. Which theoretical knowledge are the ultrasound courses supposed to impart?
(Multiple responses possible!)

- ☐ Basic physics
- ☐ Ultrasound probes
- ☐ Indications and limitations
- ☐ Advantages and disadvantages
- ☐ Anatomical structures
- ☐ Not applicable
- ☐ Other (Please name!) ________________________________

20. Which practical skills are the ultrasound courses supposed to impart?
(Multiple responses possible!)

- ☐ Choosing ultrasound probe
- ☐ Applying contact pressure and ultrasound gel
- ☐ Orientation in sagittal and transversal plane
- ☐ Giving patient instructions (e.g. breathing maneuver, change of position)
- ☐ Doppler function
- ☐ Describing findings (e.g. organ structure, echogenicity)
- ☐ Not applicable

Knobology[5]:

- ☐ Selection of patient
- ☐ Menu change
- ☐ Change of ultrasound probe
- ☐ Freeze
- ☐ Gain – overall
- ☐ Depth
- ☐ Time gain compensation
- ☐ Trackball
- ☐ Comment
- ☐ Body marker
- ☐ Measurement

☐ Other (Please name!) ________________________________

____

____

[5] Hofer, Matthias: Sono Grundkurs, pg.10.

Resources

21. Who manages <u>compulsory</u> ultrasound courses? *(Multiple responses possible!)*

Description of the course *(If not listed, please use empty cells below!)*	Clinical director	Medical Specialist	Resident	Peer-student-tutor
☐ Lecture	☐	☐	☐	☐
☐ Seminar	☐	☐	☐	☐
☐ Interdisciplinary course	☐	☐	☐	☐
☐ Laboratory course	☐	☐	☐	☐
☐ Bedside teaching	☐	☐	☐	☐
☐ Skillslab course	☐	☐	☐	☐
☐ Compulsory elective course	☐	☐	☐	☐
☐	☐	☐	☐	☐
☐	☐	☐	☐	☐

22. Who manages <u>elective</u> ultrasound courses? *(Multiple responses possible!)*

Description of the course *(If not listed, please use empty cells below!)*	Clinical director	Medical Specialist	Resident	Peer-student-tutor
☐ Lecture	☐	☐	☐	☐
☐ Seminar	☐	☐	☐	☐
☐ Skillslab course	☐	☐	☐	☐
☐	☐	☐	☐	☐
☐	☐	☐	☐	☐
☐	☐	☐	☐	☐

23. Which qualification does the teaching staff have? *(Multiple responses possible!)*

☐ DEGUM – Certificate ☐ Master of Medical Education (MME) ☐ Own didactic seminars

☐ Medical Specialist (Please specify discipline! ____________________

☐ Resident (Please specify discipline!) ____________________

☐ Not applicable ☐ Other (Please name!) ____________________

24. How many ultrasound machines are available for the education? ____________

25. Do you find education through peer-student-tutors (peer-teaching) useful?

☐ Yes ☐ No ☐ Not applicable

26. Why do you find education through peer-student-tutors (peer-teaching) useful/not useful?

Assessment methods

27. Which assessment methods are being used? *(Multiple responses possible!)*

☐ No agreement

☐ Written examination

Number of questions: ______________________________

Multiple-Choice-Questions: ☐ Yes ☐ No

Open-ended questions: ☐ Yes ☐ No

Institute for medical and pharmaceutic examination questions (IMPP)-Questions ☐ Yes ☐ No

☐ Oral examination

Number of questions: ______________________________

Number of stations: ______________________________

Duration of examination: ______________________________

Checklists for examination questions: ☐ Yes ☐ No

IMPP-Questions: ☐ Yes ☐ No

☐ Practical exam

Number of stations: ______________________________

Duration of examination: ______________________________

Number of examiners: ______________________________

Checklists for examination questions: ☐ Yes ☐ No

☐ Objective structured clinical examination (OSCE)

Number of stations: ______________________________

Duration of examination: ______________________________

Number of examiners: ______________________________

Checklists for examination questions: ☐ Yes ☐ No

☐ Other (Please name!) ______________________________

Number of stations: ______________________________

Duration of examination: ______________________________

Number of examiners: ______________________________

Checklists for examination questions: ☐ Yes ☐ No

Evaluation

28. How are the ultrasound courses evaluated?

☐ Via online questionnaire ☐ Via paper-based questionnaire ☐ Via SMS

☐ No evaluation ☐ Other (Please name!) ________________________________

29. Who conducts the evaluations?

☐ University Medical Centre ☐ Medical faculty ☐ Dean's office

☐ Students' representatives ☐ Skillslab

☐ Other ____

30. Who analyses the evaluations?

☐ University hospital ☐ Medical faculty ☐ Dean's office

☐ Students' representatives ☐ Skillslab

☐ Other ____

31. How are the evaluations published?

☐ Online ☐ Bulletin ☐ No evaluation

☐ Other ____

32. What possibilities do you see to improve the ultrasound skills education at your medical faculty/your skillslab?

Thank you for your time!
