# Supplementary material for: Undergraduate ultrasound education at German-speaking medical faculties: a survey
Source: GMS J Med Educ. 2019 Aug 15;36(4):Doc34. doi: 10.3205/zma001242 (PMC6737263; doi:10.3205/zma001242)
Supplement: Information leaflet [file JME-36-4-34-s-002.pdf]

Quantitative and qualitative situation analysis of the undergraduate ultrasound education at medial faculties in the German-speaking area

Dear Skillslabs directors in Germany, Austria and Switzerland,

Young residents are expected to have fundamental theoretical knowledge and practical skills in performing ultrasound after graduating from medical school [1]. The medical faculties of Muenster [2] and Duesseldorf [3] have published innovative teaching methods in ultrasound. However, ultrasound is not a compulsory part of the undergraduate medical curriculum and is very heterogeneously taught [4; 5].

I intend to analyse the current state of the undergraduate ultrasound education at the medical faculties in the German-speaking area and to investigate to which extent medical students get the opportunity to acquire basic skills in ultrasound. The results of this study shall be used to suggest a possible framework for a longitudinal undergraduate medical ultrasound curriculum.

Therefore, I would like to request your participation in the following survey or forwarding it on to the person responsible for ultrasound skills education at your medical faculty. Please complete the attached pdf- or word document. You may use (email address deleted) for returning documents.

For questions please contact me at any time.

[1] Subramaniam RM, Beckley V, Chan M, Chou T, Scally P. Radiology Curriculum Topics for Medical Students. Academic Radiology 2006;13:880–4. doi:10.1016/j.acra.2006.02.034.
[2] Heinzow, Hauke S.; Friederichs, Hendrik; Lenz, Philipp; Schmedt, Andre; Becker, Jan C.; Hengst, Karin et al. (2013): Teaching ultrasound in a curricular course according to certified EFSUMB standards during undergraduate medical education: a prospective study. In: BMC medical education 13, S. 84. DOI: 10.1186/1472-6920-13-84.
[3] Hofer M, Kamper L, Sadlo M, Sievers K, Heussen N. Evaluation of an OSCE assessment tool for abdominal ultrasound courses. Ultraschall Med 2011;32:184–90. doi:10.1055/s-0029-1246049.
[4] Konge, L.; Albrecht-Beste, E.; Bachmann Nielsen, M. (2015): Konge_L_Ultrasound_in_pregrad_2015 // Ultrasound in Pre-Graduate Medical Education. In: Ultraschall in der Medizin (Stuttgart, Germany : 1980) 36 (3), S. 213–215. DOI: 10.1055/s-0034-1399553.
[5] Recker, F.; Blank, V.; Diederich, H.; Huckauf, S.; Lindner, F.; Minier, M. et al. (2014): Ultraschallausbildung an deutschsprachigen Universitäten: Wo stehen wir und wo soll es hingehen? In: Ultraschall in der Medizin 35 (S 01), S. P4_12. DOI: 10.1055/s-0034-1389405.
